# Opportunities, Challenges, and Future Directions for the Integration of Automation in Nursing Practice: Discursive Study

**DOI:** 10.2196/72674

**Published:** 2025-08-14

**Authors:** Joseph Andrew Pepito, Neilan John Acaso, Rommel Merioles, Judith Ismael

**Affiliations:** 1 Department of Medical Technology, College of Allied Medical Sciences, Cebu Doctors' University, 1 Dr. P.V. Larrazabal Jr. Ave. North Reclamation, Mandaue, 6014, Philippines, 63 9984682591; 2 MultiCare Tacoma General Hospital, Tacoma, WA, United States; 3 College of Nursing, Cebu Doctors' University, Mandaue, Philippines; 4 College of Nursing and Allied Health Sciences, Cebu Institute of Technology University, Cebu, Philippines

**Keywords:** nursing automation, artificial intelligence, nursing ethics, healthcare technology, patient safety, digital health, sociotechnical systems

## Abstract

**Background:**

Global health care systems are under increasing strain due to aging populations, workforce shortages, and rising patient complexity. In response, automation technologies are being explored as a means to optimize nursing workflows, reduce burdens, and improve patient outcomes. However, the integration of such technologies raises complex ethical, legal, and professional considerations that remain insufficiently addressed in current literature.

**Objective:**

This study aims to critically examine the integration of automation into nursing practice through a discursive analysis. Specifically, it seeks to (1) identify nursing tasks most amenable to automation; (2) evaluate the benefits and drawbacks of automating these tasks; (3) explore ethical and legal implications; (4) propose strategies for ethical and equitable integration; and (5) outline future directions for research, practice, and policy.

**Methods:**

An integrative review and conceptual analysis were conducted, grounded in sociotechnical systems theory and the ethics of care. A structured search across PubMed, CINAHL, Scopus, Web of Science, and JMIR Publications identified 73 peer-reviewed papers published between 2019 and 2025. Thematic synthesis was performed to identify key domains relevant to automation in nursing.

**Results:**

Five major categories of automatable nursing tasks were identified: administrative documentation, medication management, patient monitoring, infection control, and mobility support. Automation in these areas was associated with improved efficiency, enhanced patient safety, and reduced physical and cognitive workload for nurses. Nevertheless, challenges such as deskilling, dehumanization of care, inequitable access, and unclear legal accountability were prominent. The study proposes the Integration of Automation Technologies in Nursing Practice Conceptual Framework.

**Conclusions:**

The ethical integration of automation into nursing practice requires more than technological readiness; it demands policy development, targeted education, and inclusive governance. When guided by professional values and human-centered design, automation can complement nursing practice and improve care delivery. Future research should prioritize longitudinal impact assessments, legal clarity, and equitable infrastructure investment to support sustainable adoption.

## Introduction

### Background

Health care systems worldwide face escalating demands due to aging populations, increased prevalence of chronic disease, and workforce shortages [1]. The evolving health care landscape, characterized by increasing patient complexity, health care worker shortages, and escalating health care costs, necessitates innovative approaches to optimize patient care delivery [2]. Nursing, a cornerstone of patient care, is particularly affected. Automation has emerged as a promising solution to streamline tasks, reduce errors, and enhance job satisfaction [3]. One such approach involves the automation of routine and repetitive tasks within nursing practice. However, understanding which tasks are automatable and the consequences of such automation remains underexplored. In recent years, the integration of automation technologies into health care has generated significant discourse across academic, professional, and policy-making domains [4,5]. Within the nursing profession, the adoption of automation has been hailed as a means to alleviate the workforce burden, enhance efficiency, and improve patient outcomes [6].

While technology has infiltrated various sectors, its integration within nursing has been a subject of ongoing debate and exploration. Nevertheless, the integration of these technologies presents both opportunities and challenges, warranting a nuanced examination of their implications for nursing practice. Current literature highlights the potential of automation to streamline repetitive, time-intensive tasks such as medication dispensing, patient monitoring, and administrative documentation, thereby freeing up nurses to focus on higher-level cognitive functions such as patient assessment, critical thinking, and emotional support [7].

Despite these advances, critical gaps remain in understanding the ethical, practical, and sociocultural dimensions of automating certain tasks in nursing. For instance, while some studies have emphasized the potential for task automation to reduce burnout and improve job satisfaction, others have raised concerns about deskilling, reduced human interaction, and inequities in access to technological resources [8]. The literature also reveals significant gaps in our understanding regarding patient safety, ethical considerations, job displacement, and the potential for dehumanization of care [9,10]. Existing studies primarily focus on specific technologies such as robotics or telemedicine, with limited comprehensive frameworks for evaluating the broader implications of automation within the nursing profession [7,11].

The significance of this research lies in its potential to address these gaps by providing a comprehensive exploration of the integration of automation in nursing practice, along with its associated benefits and inherent challenges. In doing so, this study seeks to inform evidence-based strategies for integrating automation into nursing workflows in a manner that aligns with professional values and enhances health care delivery. Specifically, this discursive paper aims to answer the following questions: (1) What tasks in nursing practice are most amenable to automation? (2) What are the perceived benefits and drawbacks of automating these tasks? (3) What are the ethical considerations and legal implications of automating nursing tasks? (4) How can automation be integrated ethically and equitably to maximize its positive impact while mitigating potential risks? and (5) What are the future directions for the development and integration of automated technologies in nursing?

The rationale behind this research stems from the growing demand for health care services amid an aging population, nursing shortages, and escalating care complexities. Automation, if implemented thoughtfully, holds promise as a transformative solution to these systemic challenges [12,13]. By addressing the identified research questions, this study aims to advance scholarly understanding of how automation can be leveraged in nursing practice to achieve both operational efficiency and high-quality care. Moreover, the findings have the potential to contribute to policy development, workforce planning, and the design of technology that supports, rather than supplants, the role of nurses. Ultimately, this research seeks to provide actionable insights that bridge the gap between technological innovation and the enduring humanistic ethos of nursing, ensuring that automation serves as a tool for empowerment rather than displacement. To enhance clarity and reader engagement, this paper includes visual aids, such as a table summarizing automatable nursing tasks, benefits, and ethical considerations, as well as conceptual figures presenting the integration of automation within nursing workflows.

### Thesis Statement

While automation holds promise for improving nursing efficiency and patient outcomes, this paper argues that its integration must be critically navigated to preserve ethical standards, professional integrity, and the relational core of nursing care. Through discursive analysis, the paper proposes frameworks to ensure that technological innovation supports, rather than displaces, the humanistic foundations of nursing.

## Methods

### Study Design

This discursive paper used an integrative review combined with conceptual analysis to explore the integration of automation into nursing practice. Consistent with discursive inquiry methodologies, the approach emphasized critical reflection, synthesis, and argumentation rather than empirical measurement. The study aimed to develop a theoretically informed understanding of how automation shapes nursing roles, ethics, and care delivery. Methodological rigor was ensured through a systematic literature search strategy, thematic categorization, and the application of established theoretical frameworks that guided the analysis.

### Theoretical Framework

The conceptual analysis was grounded in sociotechnical systems theory and ethics of care principles. Sociotechnical theory [14] recognizes the interdependence between social (human) and technical (automation) elements in complex systems, while the ethics of care foregrounds the relational, moral dimensions central to nursing practice [15]. These frameworks informed the identification of core concepts and the interpretation of findings, ensuring alignment with nursing’s humanistic values.

### Literature Search Strategy

A comprehensive literature search was conducted across PubMed, CINAHL, Scopus, Web of Science, and JMIR Publications to identify relevant peer-reviewed papers published between April 2019 and March 2025. The search strategy used Boolean operators and a combination of controlled vocabulary (eg, Medical Subject Headings terms) and keywords to capture a wide range of relevant literature. The key search terms included “automation in nursing,” “artificial intelligence in health care,” “robotics in nursing,” “automated decision support,” and “nursing informatics.” This approach ensured a comprehensive retrieval of studies addressing both practical implementations and theoretical frameworks related to automation in nursing. To maintain breadth and depth in the analysis, the search included both empirical studies and theoretical or discursive works.

### Inclusion and Exclusion Criteria

Studies were included in the analysis if they focused explicitly on the application, impact, or perception of automation technologies within nursing practice. In addition, eligible studies addressed ethical, legal, operational, or sociocultural dimensions of automation, providing a comprehensive view of its implications in clinical contexts. Only studies that were peer-reviewed and published in English were considered to ensure quality and accessibility.

Conversely, studies were excluded if they focused solely on disciplines outside of nursing without clear relevance to the profession. Papers that lacked sufficient methodological detail or a solid theoretical foundation were also omitted, as were opinion pieces that did not present substantive evidence or analysis.

Following the initial database search, which yielded 136 papers, a total of 73 studies met the inclusion criteria after a rigorous process of title and abstract screening, followed by full-text review.

### Data Extraction and Analytic Process

Key information was extracted using a standardized thematic framework aligned with the study’s overarching objectives. The analysis focused on five primary domains: types of automatable nursing tasks, perceived benefits and drawbacks, ethical and legal implications, strategies for ethical and equitable integration, and future research directions. These thematic categories were selected to reflect the conceptual structure of the study and guide a comprehensive synthesis of the literature.

An iterative process of inductive and deductive thematic analysis was used. The process began with open coding of the selected literature, which produced a broad set of descriptive codes. This initial coding allowed for the organic emergence of themes directly from the data. The resulting codes were then organized into thematic categories that were both informed by the study’s research objectives and theoretically anchored in sociotechnical systems theory and the ethics of care framework.

To ensure thematic coherence and analytic saturation, each emerging theme was critically appraised based on its recurrence across sources, its relevance to theoretical constructs, and its explanatory power about the study’s aims. Although the literature supported a wide range of potential themes, final theme selection was guided by their alignment with the 5-core research aims and their frequency and conceptual significance across the dataset. This rigorous and reflective synthesis enabled the condensation of a complex, multidisciplinary literature base into 5 analytically robust and practically relevant thematic domains.

## Results

### Overview

The analysis yielded 5 core thematic domains representing key areas in which automation is transforming nursing practice. These domains include (1) types of automatable nursing tasks, (2) perceived benefits and drawbacks of automation in nursing, (3) ethical and legal implications, (4) strategies for ethical and equitable integration, and (5) future directions and research gaps.

### Types of Automatable Nursing Tasks

The integration of automation technologies into nursing practice offers transformative opportunities across multiple domains of patient care and operational workflows [16]. A synthesis of the current literature reveals five primary categories of nursing tasks that are highly amenable to automation: administrative and documentation duties, medication administration and management, patient monitoring and data collection, infection control and environmental management, and patient assistance and mobility support. Automation in these areas promises to alleviate workload, enhance patient safety, and optimize health care delivery, provided that it is implemented thoughtfully.

One major area of opportunity is the automation of routine administrative tasks, which constitute a significant portion of nurses’ daily workload [6]. Technologies such as electronic health records (EHRs) streamline data entry, retrieval, and sharing, facilitating faster documentation and information exchange [17]. Automated scheduling systems optimize shift planning by balancing staff availability with patient needs [18], while voice-to-text transcription tools reduce the time nurses spend on manual charting [19]. By automating administrative responsibilities, health care organizations can free up nurses to focus on direct patient care, thereby improving both job satisfaction and care quality. For instance, at the Mayo Clinic, the integration of speech recognition tools reduced nursing documentation time by 40%, enhancing direct patient interaction [20]. Similarly, Fraser Health Authority reported a 91% accuracy in scheduling after adopting artificial intelligence (AI)-based workforce planning software [21].

Equally critical is the automation of medication administration and management, where precision and reliability are paramount. Automated dispensing cabinets, such as Pyxis (Pyxis Corporation) and Omnicell (Omnicell, Inc), enhance medication security, reduce errors, and provide real-time inventory tracking [22]. Smart infusion pumps equipped with dose error reduction systems proactively alert clinicians to potential dosing mistakes based on preprogrammed limits [23]. A 2022 multihospital study at the Mayo Clinic observed a 16% reduction in medication errors following the implementation of smart infusion pumps with integrated dose error reduction systems [24]. Robotics further augments this domain by automating medication preparation and bedside delivery; for instance, TUG robots at Dartmouth Hitchcock Medical Center autonomously navigate clinical environments to deliver medications securely, reducing medication delivery times and improving workflow efficiency [25]. Integrating these technologies with EHRs ensures seamless prescription tracking and communication, collectively minimizing the risk of adverse drug events.

Building on these innovations, automation has significantly advanced patient monitoring and data collection. Continuous glucose monitors and telemetry devices allow for real-time tracking of vital signs, enabling early detection of physiological deterioration [26]. Wearable biometric sensors support remote monitoring of chronic conditions, leading to reductions in hospital readmissions [27]. Johns Hopkins University demonstrated the efficacy of wearable biosensors and predictive analytics for sepsis detection, resulting in an 18.2% decrease in sepsis-related mortality [28]. The wearable health patch used for chronic obstructive pulmonary disease monitoring enabled continuous, remote physiological data collection, supporting early detection and lowering hospital readmission rates [29]. Internet of Things–integrated monitoring systems facilitate continuous data capture and analysis, reducing manual errors and enhancing the accuracy of clinical decision-making [30].

In the realm of infection control and environmental management, automation serves as a critical ally in minimizing health care–associated infections. Technologies such as UV-C disinfection robots and hydrogen peroxide vapor systems have been shown to substantially reduce bacterial contamination in clinical environments [31]. At an academic hospital in Austria, UV-C disinfection robots led to a 96.9% reduction in bacterial surface contamination, contributing to lower infection rates [32]. Automated hand hygiene monitoring systems, using radio-frequency identification and video analytics, enhance compliance among health care workers [33]. Radio-frequency identification–enabled hand hygiene compliance systems at Toronto General Hospital boosted adherence by 38%, demonstrating the potential of automated surveillance to reinforce institutional infection control protocols. In addition, AI-driven surveillance platforms improve early outbreak detection by analyzing EHR data for unusual infection patterns [34]. Robotic waste management systems and smart bins further reduce contamination risks by optimizing hazardous material handling and disposal processes [35].

Finally, the automation of patient assistance and mobility support addresses both patient safety and the physical demands placed on nurses. Robotic lifts and transfer devices assist with repositioning and mobility tasks, mitigating the risk of musculoskeletal injuries among staff [35]. A scoping review reported reductions in musculoskeletal injuries following the implementation of robotic exoskeletons, alongside improved patient rehabilitation outcomes [36]. Smart beds equipped with pressure sensors and automated repositioning features not only improve patient comfort but also help prevent pressure ulcers [37]. In long-term care settings, smart beds with pressure-sensing and repositioning features have reduced the incidence of pressure ulcers, improving patient comfort while reducing physical demands on nursing staff. Robotic exoskeletons support patients with mobility impairments during rehabilitation, promoting independence while reducing nurse burden [38].

In summary, the types of nursing tasks most amenable to automation are those characterized by routine, repetitive, or high-risk activities in which technology can reliably enhance efficiency, safety, and care outcomes. Administrative workflows, medication management, patient monitoring, infection control, and mobility assistance each present a compelling case for automation, backed by a growing body of empirical evidence. By strategically adopting these technologies, health care systems can optimize nursing workflows while safeguarding the humanistic core of the nursing profession. A balanced, evidence-based integration of automation, therefore, holds the potential to transform nursing practice while preserving its essential values (Table 1).

**Table 1.** Summary of automation domains, associated technologies, benefits, and ethical implications in nursing practice.

Domain	Example technologies	Benefits	Ethical or legal considerations
Administrative tasks and documentation	Scheduling tools, automated check-in kiosks, voice-to-text systems, and smart charting software	Reduced documentation burden, improved workflow, and enhanced data accuracy	Privacy concerns, documentation integrity, and risk of deskilling
Medication management	Automated dispensing cabinets, barcode scanning, and smart infusion pumps	Reduced medication errors, improved safety, and time efficiency	Overreliance on automation and liability in medication errors
Patient monitoring	Wearable sensors and AI-driven^a^ predictive analytics dashboards	Early detection of deterioration and fewer ICU^b^ admissions	Algorithmic bias, data accuracy, and alarm fatigue
Infection control	UV disinfection robots and automated hand hygiene monitoring systems	Reduced hospital-acquired infections and improved compliance	Accountability for failure and surveillance ethics
Patient mobilization	Robotic lifts and exoskeletons	Fewer musculoskeletal injuries and safer patient transfers	Informed consent and device malfunction

a AI: artificial intelligence.

b ICU: intensive care unit.

### Benefits and Drawbacks of Automation in Nursing

While automation introduces significant advantages—such as reduced workload, enhanced safety, and streamlined workflows—it also presents critical challenges that must be thoughtfully managed to avoid unintended consequences. Several benefits discussed earlier, including improved efficiency in documentation, medication accuracy, and real-time monitoring, are being realized in frontline care. However, to maintain coherence with the overarching themes of this paper, it is equally essential to examine the nuanced drawbacks and how these are being addressed in practice.

A primary concern is the dehumanization of care, particularly when routine interactions are delegated to machines [10]. For example, while robotic medication carts can enhance efficiency, studies show that patients sometimes perceive these interactions as impersonal, reducing their sense of emotional support and trust [39]. This aligns with the broader ethical theme of preserving relational care in the face of technological delegation.

Another key drawback is the risk of deskilling. As automation assumes responsibility for routine tasks, such as documentation via voice-to-text or algorithm-driven triage, there is concern that nurses may lose proficiency in essential clinical and cognitive skills over time [40]. This risk is particularly salient among early-career nurses, who may rely more heavily on automated systems [41]. To counteract this, some institutions have introduced hybrid training models that pair manual and automated workflows, reinforcing foundational competencies while promoting digital fluency [42].

Equitable access is also a pressing issue. Rural or under-resourced facilities may lack the infrastructure to adopt sophisticated automation technologies, exacerbating health care disparities [43]. For instance, while smart beds and robotic lifts are transforming mobility assistance in urban hospitals, facilities in remote regions continue to rely on manual methods. Policy makers and health care systems must prioritize equitable technology distribution and workforce support to prevent systemic gaps.

Real-life implementations further illustrate how health care settings are navigating this balance. At Mount Sinai Hospital, the integration of predictive analytics for early sepsis detection has reduced ICU admissions by 23%. However, the hospital also instituted a “human-in-the-loop” protocol requiring nurse verification of all algorithm-based alerts, ensuring that clinical judgment is not bypassed [44]. Similarly, at Tallahassee Memorial HealthCare, robotic exoskeletons have been adopted to assist in patient mobility but are always deployed alongside staff to preserve patient-nurse interaction and ensure safety [45].

Ultimately, the integration of automation in nursing must be guided by deliberate strategies that align technological efficiency with professional integrity and ethical care. Continuous evaluation, inclusive co-design, and hybrid implementation models represent practical approaches to achieving a sustainable balance between automation’s benefits and its potential drawbacks.

### Ethical and Legal Implications

#### Overview

The integration of automation into nursing practice presents both significant promise and profound ethical and legal challenges. The current ethical and legal landscape in this domain is fragmented, characterized by evolving policies, emerging frameworks, and limited case law specific to nursing [16,46]. While foundational principles such as patient autonomy, data privacy, and informed consent are well established within health care ethics, their application to AI-driven or automated nursing tasks remains underdeveloped in both policy and practice [47,48].

#### Ethical Landscape: Established and Emerging Areas

On the ethical front, principles such as autonomy, beneficence, nonmaleficence, and justice remain cornerstones. However, their operationalization within automated systems often lacks clarity [49]. For example, while nurses are ethically obliged to respect patient autonomy, the use of algorithmic decision support tools can inadvertently standardize care in ways that diminish individualized, contextualized decision-making [50]. Similarly, concerns about algorithmic bias, such as underrepresentation of minority populations in training datasets, pose equity challenges that current ethical frameworks only partially address [51,52].

Practical initiatives addressing these concerns are beginning to emerge. At institutions such as Johns Hopkins and Kaiser Permanente, AI toolkits are being codeveloped with nurses to evaluate fairness and transparency during pilot phases, embedding ethical scrutiny into design and deployment processes [53]. These models exemplify how ethics can be embedded at the system level rather than imposed retroactively.

#### Legal Landscape: Gaps and Opportunities

Legally, the terrain is less defined. While privacy regulations such as the Health Insurance Portability and Accountability Act (United States) and the General Data Protection Regulation (European Union) provide a baseline for data protection, there is little consensus on liability in cases where automated systems contribute to patient harm [54,55]. Questions remain about whether responsibility lies with nurses, software developers, or health care institutions when AI malfunctions lead to adverse outcomes. Although regulatory bodies such as the US Food and Drug Administration (FDA) have begun classifying and approving AI-driven clinical tools [56,57], these often focus on static algorithms and offer limited guidance for dynamic, learning systems that evolve postdeployment.

In contrast, the European Union’s AI Act represents a forward-looking model by categorizing health care AI as “high-risk” and mandating transparency, human oversight, and postmarket surveillance [58]. However, practical implementation remains at an early stage, and nursing-specific legal guidance is still lacking.

#### From Concept to Practice: Grounding Ethical-Legal Integration

While previous discussions often remain at a conceptual level, actionable pathways are necessary to ensure that automation supports nursing values rather than undermining them. A balanced approach integrating ethical guidelines and legal safeguards must be grounded in concrete practices such as mandatory ethical impact assessments before implementation of automation tools, focusing on issues such as bias, accessibility, and implications for patient-nurse interaction; clear role delineation and documentation protocols that define when nurses may override AI outputs and how such decisions should be recorded, thereby preserving clinical judgment and legal clarity; ethics and legal literacy programs, integrated into continuing professional development, to equip nurses with the knowledge to navigate emerging automation technologies responsibly; pilot testing with human-in-the-loop models, such as requiring nurse validation before automated triage decisions are enacted, already in use at several US trauma centers, to ensure accountability and foster trust.

In conclusion, while the ethical and legal frameworks governing automation in nursing are still maturing, incremental steps toward embedding these considerations into technology design, policy development, and clinical education are both feasible and necessary. Grounding these principles in practical strategies can help ensure that automation is not only safe and effective but also aligned with the core values of nursing care.

### Strategies for Ethical and Equitable Integration

As automation increasingly permeates health care systems, its integration into nursing practice must be approached with deliberate, ethical, and equity-focused strategies. The goal is not merely to introduce technology for operational efficiency, but to align automation with the core values of the nursing profession—compassion, advocacy, and patient-centered care—while also addressing the risk of exacerbating existing health disparities and workforce inequalities.

#### Ethical Principles in Automation Integration

Foundational ethical principles of autonomy, beneficence, nonmaleficence, and justice must underpin the deployment of automation technologies in nursing practice [59]. Studies underscore the potential of automation to enhance nursing efficiency, accuracy, and safety, particularly in routine, repetitive, and data-intensive tasks such as medication administration, documentation, and vital signs monitoring. However, automation must not compromise the nurse-patient relationship or marginalize vulnerable populations.

Autonomy is particularly relevant where AI-driven decision support tools are deployed; nurses must retain clinical judgment and the ability to override automated recommendations when they conflict with contextual patient needs [60]. Similarly, beneficence and nonmaleficence demand that technologies demonstrably improve patient outcomes and do not introduce new harms, whether physical, emotional, or relational [61]. Justice, in turn, mandates equitable access to the benefits of automation across diverse patient populations and care settings [62].

#### Addressing Equity in Technological Design and Deployment

Current research emphasizes that digital health innovations often reflect and reinforce systemic inequities when implemented without inclusive design. Therefore, strategies for equitable implementation should begin at the design stage. Inclusive co-design approaches, such as engaging nurses from diverse backgrounds, patients, and other stakeholders, can help ensure that automated systems are culturally sensitive, linguistically appropriate, and attuned to the unique needs and contexts of underrepresented populations. Moreover, rural and resource-limited settings must be considered in automation planning. Without equitable infrastructure investments, such areas risk being left behind in the digital transformation of care, further widening health outcome gaps.

#### Workforce Implications and Professional Integrity

Ethical implementation also requires thoughtful consideration of the nursing workforce. Automation should be framed not as a replacement, but as a tool that augments nursing capabilities. Evidence suggests that when automation is introduced transparently and with robust change management strategies, it can reduce cognitive burden and allow nurses to focus on complex, relational aspects of care [3,8]. However, there is also evidence of skepticism and resistance when automation is perceived as a threat to job security or professional identity.

To address these concerns, health care organizations must invest in continuous professional development, ensuring that nurses are not only technologically competent but also empowered to contribute to implementation decisions [63,64]. This participatory approach respects nurses’ experiential knowledge and fosters a culture of trust, critical for successful technology adoption.

#### Governance, Accountability, and Legal Considerations

Effective governance mechanisms are essential to oversee the ethical use of automation in nursing [55,65]. This includes establishing clear lines of accountability, particularly in scenarios involving clinical decision support systems or autonomous robotics. The absence of well-defined legal and regulatory frameworks poses challenges in attributing responsibility for errors or adverse outcomes arising from automated actions.

**Figure 1.**
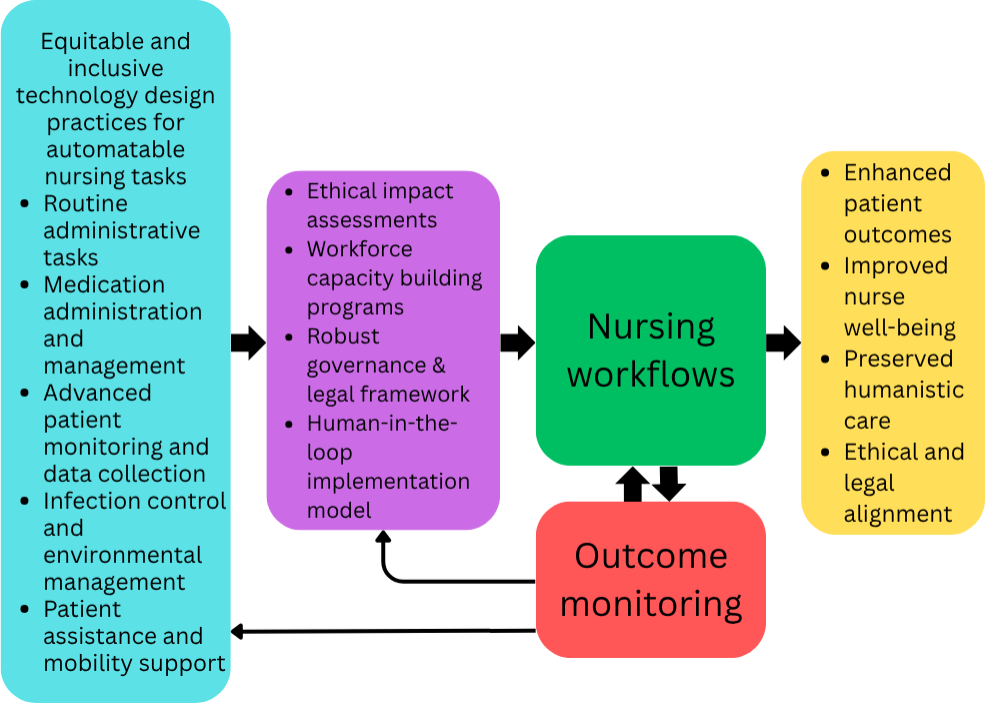
The Integration of Automation Technologies in Nursing Practice Conceptual Framework illustrates the integration of automation technologies into nursing practice by linking equitable and inclusive technology design for automatable tasks with nursing workflows, guided by ethical governance and human-centered implementation. It positions ethical impact assessments, workforce development, and legal frameworks as mediating structures that ensure automation supports nursing workflows without compromising care quality. Outcome monitoring feeds back into both design and governance, creating a continuous improvement loop. This framework aligns with the study’s research questions by mapping how ethical, technical, and organizational factors interact to optimize nurse well-being, preserve humanistic care, and enhance patient outcomes.

Professional bodies and regulatory agencies must collaborate to develop standards that protect both patients and practitioners, ensuring that automation enhances rather than undermines professional accountability [55,66]. Policies must also address data privacy, algorithmic transparency, and bias mitigation in AI systems used in nursing care. The Integration of Automation Technologies in Nursing Practice Conceptual Framework illustrates the integration of automation technologies into nursing practice in Figure 1. 

### Future Directions and Research Gaps

To ensure the ethical, effective, and equitable integration of automation into nursing practice, future work must align with the core thematic domains identified in this study. The following narrative outlines tailored research, practice, and policy directions under each theme to guide meaningful advancement in the field.

#### Automatable Nursing Tasks: Refinement and Validation

Future research should focus on identifying context-specific opportunities for automation using mixed methods that assess task suitability across different care environments, such as long-term and acute care settings. On the practical front, pilot studies can evaluate automation’s effectiveness in emerging domains, such as discharge planning or clinical triage, while simultaneously measuring impacts on workflow and patient experience. From a policy perspective, the development of standardized evaluation frameworks will be critical to support regulatory bodies and institutions in making informed, evidence-based decisions aligned with nursing priorities.

#### Benefits and Drawbacks: Longitudinal Impact Assessment

Research in this area should include long-term studies that assess automation’s influence on nurse burnout, job satisfaction, and patient outcomes, while also addressing potential unintended consequences such as deskilling or the depersonalization of care. In clinical practice, the use of human-centered metrics—such as patient-nurse trust and perceived empathy—alongside traditional indicators can provide a more holistic understanding of automation’s value. Policy efforts should mandate outcome monitoring and the incorporation of fail-safe mechanisms, including human-in-the-loop design and regular reassessment protocols to ensure that automation remains accountable to patient and nurse well-being.

#### Ethical and Legal Implications: Translating Principles Into Practice

Future research should aim to operationalize abstract ethical concepts, such as autonomy, beneficence, and justice, by transforming them into measurable safeguards embedded within automated systems. In practice, the implementation of ethical impact assessments before technology adoption should be normalized, with nurse involvement in co-design and governance structures that enhance transparency and accountability. On the policy front, legal frameworks must evolve to address liability concerns associated with automation. National regulations should adopt and localize global standards, such as the EU AI Act, to establish clear legal guidance tailored to specific nursing contexts.

#### Implementation Strategies: Inclusive and Adaptive Models

Research should explore best practices in co-design, involving not only nurses but also patients and underrepresented groups, to improve the cultural fit, usability, and safety of automation tools. In the clinical realm, hybrid implementation models—which combine automation with manual oversight—should be tested and refined for efficacy, equity, and resilience across diverse care settings. Policy measures should support resource allocation strategies that enable adoption in rural and under-resourced institutions, thereby mitigating the risk of deepening the digital divide in nursing care delivery.

#### Education, Workforce, and Policy Alignment: Capacity Building

Research should evaluate how digital literacy, AI ethics, and interdisciplinary competencies are taught in nursing education, with attention to gaps in confidence and skill development. From an educational standpoint, nursing curricula must be updated to integrate informatics, AI governance, and collaborative learning, ensuring that nurses are well-prepared to lead in increasingly tech-integrated environments. At the policy level, regulatory and licensing bodies should establish and enforce core competencies for nurses working with automation. These efforts should be supported by the development of credentialing pathways and continuing education mandates that promote ongoing capacity building.

By aligning future directions with the themes explored in this paper, the nursing profession can ensure that automation evolves in ways that are ethically responsible, clinically effective, and socially equitable. Through focused efforts across research, practice, and policy domains, nursing can shape the integration of automation as a tool for improving care—rather than diminishing it.

## Discussion

### Implications for Nursing Practice

The integration of automation into nursing practice represents a transformative shift in health care delivery. It offers significant potential to enhance efficiency, optimize patient outcomes, and support workforce sustainability. However, realizing these benefits requires deliberate, evidence-based strategies for practice adaptation, professional development, and system redesign. Several actionable implications emerge from the synthesis of current literature and analysis.

### Integration of Digital Literacy in Nursing Curricula

To adequately prepare future nurses for a technologically enhanced health care environment, nursing education must evolve to incorporate comprehensive digital literacy components. Digital literacy should extend beyond basic computer skills to encompass competencies in health informatics, AI ethics, algorithmic decision-making, cybersecurity, and data privacy principles [67].

Specifically, nursing curricula should embed mandatory courses on health information technology and AI in health care, covering subjects such as the operation of clinical decision support systems, the risks associated with algorithmic bias, and the principles of data stewardship [68,69]. In addition, simulation-based training should be used, allowing students to interact with AI-driven tools—such as predictive analytics dashboards and automated medication dispensers—to promote critical thinking around technology-supported clinical decision-making [70,71]. Furthermore, interprofessional education modules should be introduced, where nursing students collaborate with informaticians, engineers, and ethicists to solve clinical scenarios involving automation, fostering both technical fluency and collaborative problem-solving skills [72,73]. These curricular innovations can ensure that newly qualified nurses possess the digital fluency required to safely, ethically, and effectively engage with automated systems in clinical settings.

### Competency Framework for Human-AI Collaboration

The successful integration of automation in nursing practice requires the development of a structured competency framework for human-AI collaboration. Building on emerging models in medical informatics and AI governance, this framework should define key competencies for nurses in several critical areas.

Nurses must develop the ability to critically evaluate AI outputs, including assessing the validity, reliability, and clinical relevance of AI-generated recommendations [74,75]. Ethical and legal reasoning is also essential; nurses should be well-versed in the ethical frameworks and regulatory guidelines that govern the use of automated technologies in clinical care [55,76]. Moreover, nurses should be trained in clinical judgment augmentation, synthesizing AI insights with holistic patient assessments to inform decisions, rather than relying on technology to dictate care [77,78]. Another vital area is advocacy and patient education, where nurses must be equipped to advocate for patients when automation introduces risk or confusion, and to educate patients about the role of technology in their care [55,79]. Finally, nurses should be able to navigate health care systems and report incidents, demonstrating awareness of automation-related failures and the protocols for escalating such issues within institutional frameworks [3].

These competencies can be operationalized by health care institutions and professional organizations through the creation of credentialing pathways, the provision of continuing education programs, and the use of competency-based assessments to ensure readiness for AI-integrated practice.

### Concrete Applications: Automation Improving Outcomes and Reducing Workload

Real-world examples from clinical settings underscore the potential of automation to enhance both outcomes and workload management in nursing.

One such example is the early detection of patient deterioration through AI-driven predictive analytics. These systems have been shown to reduce unplanned ICU admissions by 25% by providing real-time alerts to nurses regarding changes in patient status. This enables timely escalation of care and improves patient safety [16].

In the area of medication management, the use of automated dispensing cabinets has led to a 30% reduction in medication errors, along with decreased narcotic diversion rates and minimized time spent on medication rounds. These efficiencies allow nurses to devote more time to direct patient care and patient education [80].

In addition, robotic assistance in patient mobilization—such as robotic exoskeletons and smart lift devices—has significantly lowered the incidence of musculoskeletal injuries among nurses by reducing physical strain during patient transfers. This not only protects the well-being of nursing staff but also improves patient safety through safer and more efficient mobilization practices [45].

Collectively, these examples demonstrate that when thoughtfully implemented, automation can serve as a powerful tool to support clinical efficiency, nurse well-being, and patient-centered outcomes.

### Conclusion

The integration of automation into nursing practice represents a paradigm shift with the potential to transform health care delivery by enhancing operational efficiency, reducing cognitive and physical burden, and improving patient outcomes. This paper has identified five key domains of automatable nursing tasks: administrative documentation, medication management, patient monitoring, infection control, and mobility assistance, each demonstrating substantial promise for streamlining workflows and enabling nurses to focus on relational, analytical, and human-centered care.

However, automation is not a neutral intervention; its implementation raises complex ethical, legal, and sociotechnical challenges that, if unaddressed, may compromise the foundational values of nursing. The findings emphasize that the humanistic core of nursing—embodied in empathy, critical judgment, and person-centered engagement—must remain central as automation becomes increasingly embedded in clinical settings. Multimedia Appendix 1 depicts a graphical abstract summarizing these findings.

To guide the practical and ethical implementation of automation in nursing, we offer the following prioritized, actionable recommendations for stakeholders, including policy makers, educators, health care leaders, and technology developers. First, it is important to establish robust governance and legal frameworks. This involves developing comprehensive regulatory policies that delineate liability, ensure algorithmic transparency, and safeguard data privacy. National nursing boards and health authorities should collaborate with legal experts and technologists to create adaptive, profession-specific guidelines that anticipate the evolving capabilities of AI and automation systems. Second, stakeholders should implement workforce capacity-building programs by integrating digital health literacy, ethics of automation, and informatics competencies into nursing education and continuing professional development. Institutions should adopt a tiered training model that equips nurses at all levels with the critical skills to engage with, interpret, and, when necessary, override automated systems in clinical contexts. Third, it is essential to advance equitable and inclusive design practices. Automation technologies should be developed through participatory co-design involving frontline nurses, patients from diverse backgrounds, and underserved communities. Policy makers must prioritize infrastructure investment in rural and low-resource settings to mitigate the digital divide and promote equitable access to innovation. Fourth, human-in-the-loop implementation models should be adopted. This means deploying automation in ways that preserve and augment clinical judgment rather than replace it. This includes embedding nurse validation protocols in AI decision-making pathways, promoting hybrid workflows, and conducting ongoing assessments of automation’s impact on empathy, job satisfaction, and patient trust. Finally, there is a need to mandate ethical impact assessments and outcome monitoring. Before widespread deployment, automation tools should undergo rigorous ethical evaluation to assess potential harms, biases, and unintended consequences. Postimplementation, health care systems must continuously monitor both technical performance and relational outcomes, such as patient-nurse communication quality and perceived quality of care.

Taken together, these recommendations provide a roadmap for aligning technological advancement with the ethical imperatives and professional identity of nursing. As the health care sector moves toward increasing reliance on intelligent systems, the nursing profession must proactively shape the discourse and deployment of automation to ensure it serves as a tool for empowerment, equity, and compassionate care.

## Supplementary material

10.2196/72674 Multimedia Appendix 1 Graphical abstract.

## Abbreviations

AI: artificial intelligence
EHR: electronic health record
FDA: US Food and Drug Administration
