# Supplementary figures and images for: Opportunities, Challenges, and Future Directions for the Integration of Automation in Nursing Practice: Discursive Study
Source: JMIR Nurs. 2025 Aug 14;8:e72674. doi: 10.2196/72674 (PMC12352802; doi:10.2196/72674)

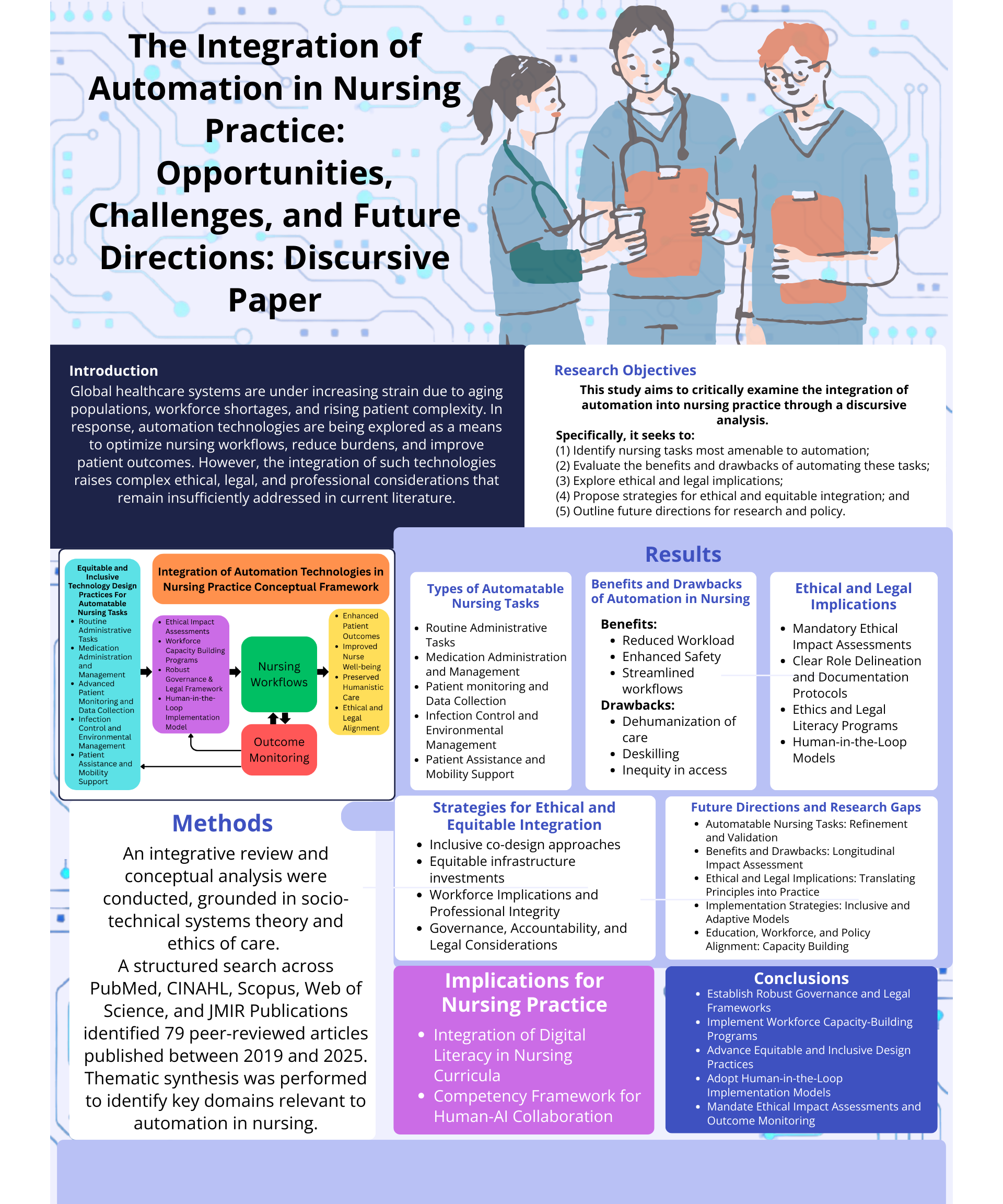

Supplement: Multimedia Appendix 1 [file nursing-v8-e72674-s001.png]
